# Significance and usefulness of imaging characteristics of gubernaculum tracts for the diagnosis of odontogenic tumors or cysts

**DOI:** 10.1371/journal.pone.0199285

**Published:** 2018-07-06

**Authors:** Masafumi Oda, Ikuko Nishida, Ikuya Miyamoto, Katsura Saeki, Tatsurou Tanaka, Shinji Kito, Noriaki Yamamoto, Naomi Yada, Daigo Yoshiga, Shinobu Matsumoto-Takeda, Nao Wakasugi-Sato, Manabu Habu, Masaaki Kodama, Shinya Kokuryo, Kenji Osawa, Shun Nishimura, Takaaki Joujima, Yuichi Miyamura, Kou Matsuo, Kazuhiro Tominaga, Izumi Yoshioka, Kenshi Maki, Yasuhiro Morimoto

**Affiliations:** 1 Division of Oral and Maxillofacial Radiology, Kyushu Dental University, Kitakyushu, Japan; 2 Division of Developmental Stomatognathic Function Science, Kyushu Dental University, Kitakyushu, Japan; 3 Division of Oral Medicine, Kyushu Dental University, Kitakyushu, Japan; 4 Department of Oral and Maxillofacial Surgery, Oita University, Oita, Japan; 5 Division of Oral Pathology, Kyushu Dental University, Kitakyushu, Japan; 6 Division of Maxillofacial Surgery, Kyushu Dental University, Kitakyushu, Japan; Northwestern University Feinberg School of Medicine, UNITED STATES

## Abstract

We aimed to determine the significance and usefulness of imaging characteristics of gubernaculum tracts (GT) for the diagnosis of odontogenic tumors or cysts. This was a retrospective analysis of relationships between odontogenic or non-odontogenic tumors or cysts and the GT that were visualized using multi-detector computed tomography (MDCT). The relationship between the size of a mass and expansion of the GT in all odontogenic tumors or cysts to which GTs were contiguous on MDCT, was statistically analyzed. Intact or expanded GTs were detected in MDCT images on the top of almost all odontogenic tumors or cysts, but not on non-odontogenic tumors or cysts. Characteristic image findings regarding the relationship between the GT and the odontogenic mass were detected for the respective odontogenic tumors or cysts in which the GTs were contiguous to the mass on MDCT. In ameloblastomas, expansion of the GTs significantly and very strongly correlated with tumor size (r = 0.741, p = 0.0001), but this correlation was very weak in dentigerous cysts (r = 0.167, p = 0.028) and there was no correlation between these parameters in odontogenic keratocysts (r = -0.089, p = 0.557). The imaging characteristics of GTs at the top of masses should be very useful for both the differential diagnosis of the pathological diagnosis of odontogenic masses and for differentiation between odontogenic and non-odontogenic masses.

## Introduction

Previously, we precisely visualized the pathway of the gubernaculum tract (GT) using cone-beam computed tomography and multi-detector computed tomography (MDCT) [1, 2]. The fibrous GT band connects the pericoronal follicular tissue of a successional tooth with the overlying gingiva and constitutes a pathway from the dental follicle to the gingiva for eruption of permanent teeth [3]. The main role of the GT is likely to be in induction of normal tooth eruption [1, 2, 4].

The GT is a canal that contains the gubernacular cord (GC). The remnants of this cord could be the basis of the development of some odontogenic tumors and/or cysts. Pathological or imaging investigations have provided strong evidence to suggest that the GC may also be the origin of adenomatoid odontogenic tumors (AOT), and that the GT might be associated with the development of odontomas [2, 5, 6].

However, as there have been few reports of the imaging characteristics of the GT, it is unclear if there is a relationship between the GT and other odontogenic masses. To investigate this possibility, in the present study, we precisely analyzed the imaging characteristics of the GT, including the spatial relationship between the GT and representative odontogenic or non-odontogenic masses on MDCT.

## Materials and methods

This study was a retrospective study of the MDCT images of 313 patients (178 males, 135 females; mean age, 43.4 years; range, 6 to 89 years), who had nine types of odontogenic and non-odontogenic masses, and who had visited the Division of Oral and Maxillofacial Radiology of Kyushu Dental University Hospital between 2009 and 2016 (Table 1). Of the patients selected with odontogenic masses, 21 cases were diagnosed as ameloblastoma (AM), 50 as odontogenic keratocyst (OKC), 2 as calcifying odontogenic cyst (COC), and 182 as dentigerous cyst (DC) by pathological examinations. Of the patients selected with non-odontogenic masses, 9 cases were diagnosed as ossifying fibroma, 1 as fibroma, 26 as nasopalatine duct cyst, 18 as simple bone cyst and 4 as schwannoma by pathological examinations. The present study was approved by the institutional review board of Kyushu Dental University (No. 14–29). In MDCT examinations, informed consent was obtained from all patients and their parents or guardians of minors by verbal.

**Table 1:** Type and gender distribution of the 313 masses in this study.

	Male	Female	Total
**Odontogenic masses**			
**Ameloblastomas**	12	9	21
**Odontogenic keratocysts**	31	19	50
**Calcifying odontogenic cysts**	-	2	2
**Dentigerous cysts**	110	72	182
**Non-odontogenic masses**			
**Ossifying fibroma**	-	9	9
**Fibromas**	-	1	1
**Nasopalatine duct cysts**	17	9	26
**Simple bone cysts**	7	11	18
**Schwannomas**	1	3	4
**Total**	178	135	313

The Activion 16 (Toshiba Co. Ltd., Tokyo, Japan) was used for MDCT. MDCT images of contiguous sections (2 mm) were taken in the axial plane to examine each region within the maxilla or/and mandible. Images were obtained with standard algorithms and bone-target windows. All CT images were analyzed using a PC-based, digital viewing system (Ziostation 2, Ziosoft, Tokyo, Japan). This analysis uses multi-planar reconstruction methods that allow the selection of appropriate slices such as axial, panoramic, or sagittal slices for comparison of images and for adaptation of the level of the gray scale in the images.

**Fig 1:**
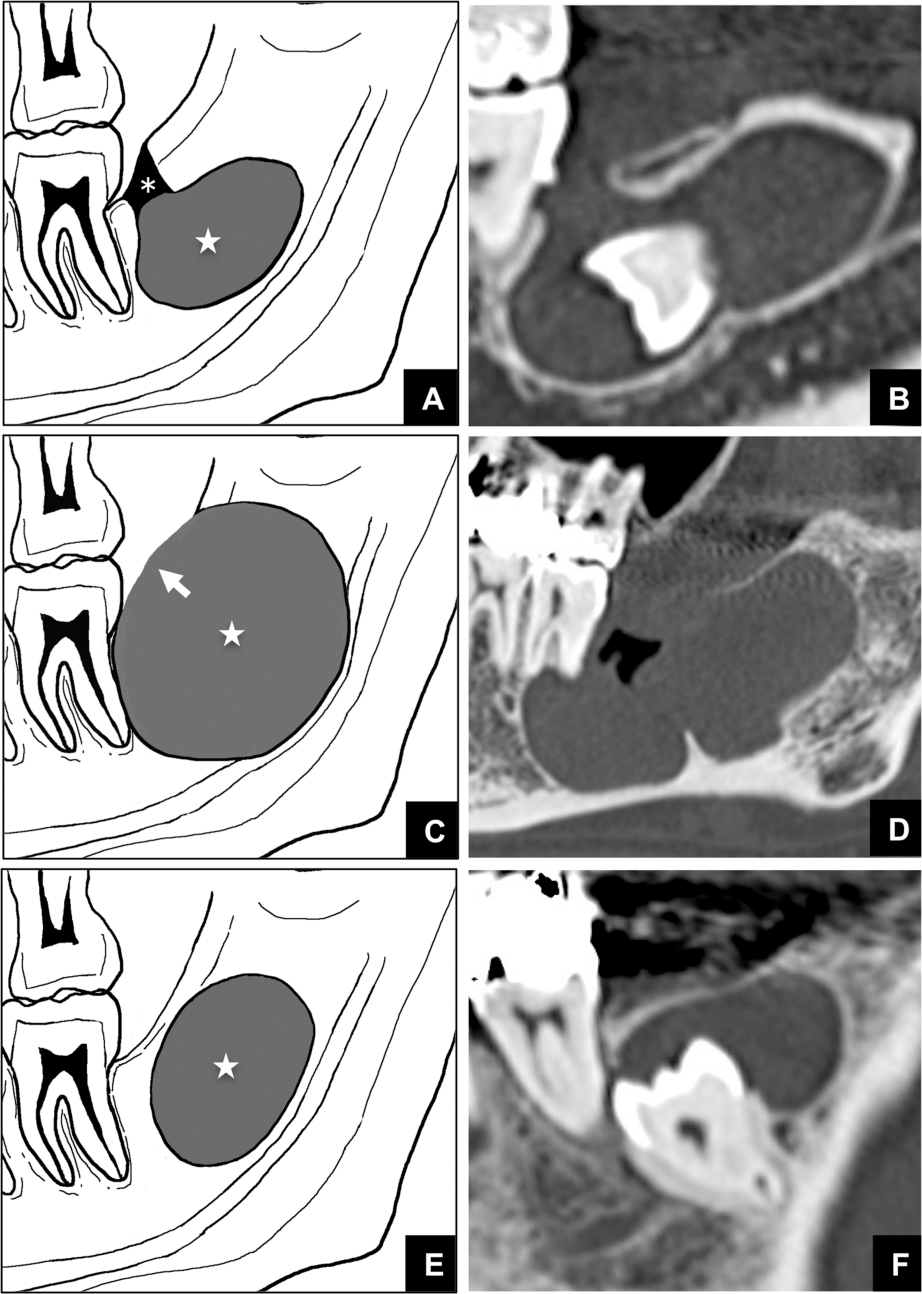
Schemes and representative MDCT images of classification into 3 groups based on the presence or absence of continuity between the GT and the top of masses in alveolar bone. **(A)** Scheme of Group 1, which was defined as cases in which an almost intact GT (asterisk) continues to the top of the mass (star). **(B)** An MDCT image of a mass classified as Group 1. (**C)** Scheme of Group 2, which was defined as cases in which an expanded GT (arrow) continues to the top of the mass (star). (**D)** An MDCT image of a mass classified as Group 2. (**E)** Scheme of Group 3, which was defined as cases in which the GT is not continuous with the tumor or cyst (star). **(F)** An MDCT image of a mass classified as Group 3.

The relationship between all the imaged masses and the GT or alveolar crest within the maxilla and mandible was precisely evaluated on MDCT. Using these MDCT data, the 313 patients were divided into 3 groups based on the continuity of the mass with the GT (Fig 1). Group 1 was defined as cases in which an almost intact GT was in contact with the tumor or cyst (Fig 1). Group 2 was defined as cases in which the area of the bone defect at the top of the alveolar bone that included a possibly expanded GT was in contact with the top of the tumor or cyst (Fig 1). Group 3 was defined as cases in which the GT was not continuous with the tumor or cyst (Fig 1). Concurrently, we measured the size of each mass by measuring the major axis of the mass and we measured the major axis of the GT or the continuity area. The continuity area was defined as the area in continuous contact with the possibly expanded GT and the bone defect areas at the top of the alveolar bone. The images were assessed by a single, experienced oral and maxillofacial radiologist (O. M.).

Statistical software, SPSS version 11 (SPSS, Chicago, IL), was used for all statistical analyses. Fisher’s exact test, t-test or Pearson’s correlation coefficient was used to compare categorical variables. A value of p<0.05 was considered to be significant.

## Results

### Odontogenic or non-odontogenic masses differ in their continuity with the GT on MDCT images

**Fig 2:**
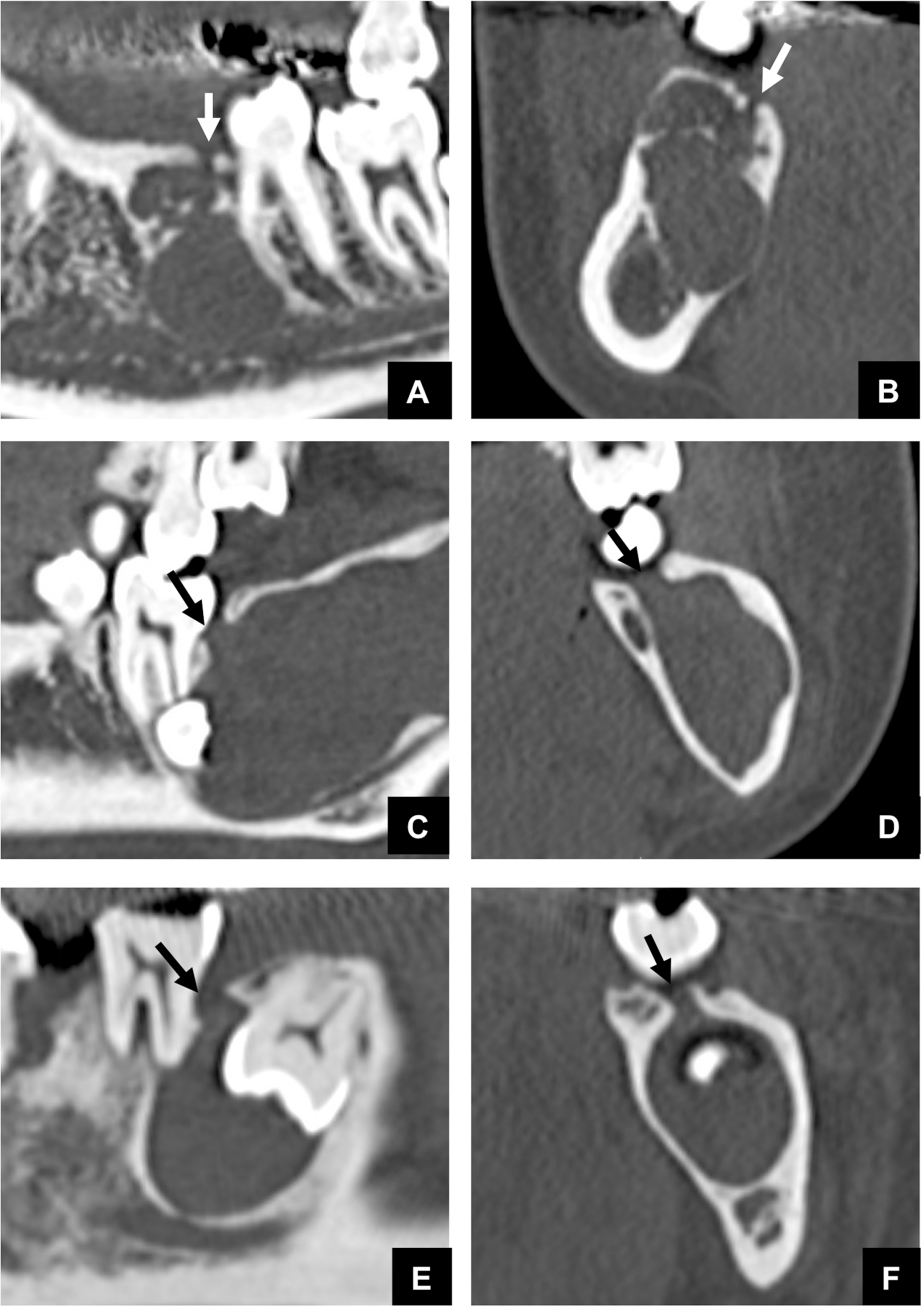
Representative MDCT images of odontogenic masses and the GT (arrows) in cases classified as Group 1. **(A)** Panoramic MDCT image of an AM case classified as Group 1. **(B)** Cross sectional MDCT image of Fig 2A. **(C)** Panoramic MDCT image of an OKC case classified as Group 1. **(D)** Cross sectional MDCT image of Fig 2C. **(E)** Panoramic MDCT image of a DC case classified as Group 1. **(F)** Cross sectional MDCT image of Fig 2E.

**Fig 3:**
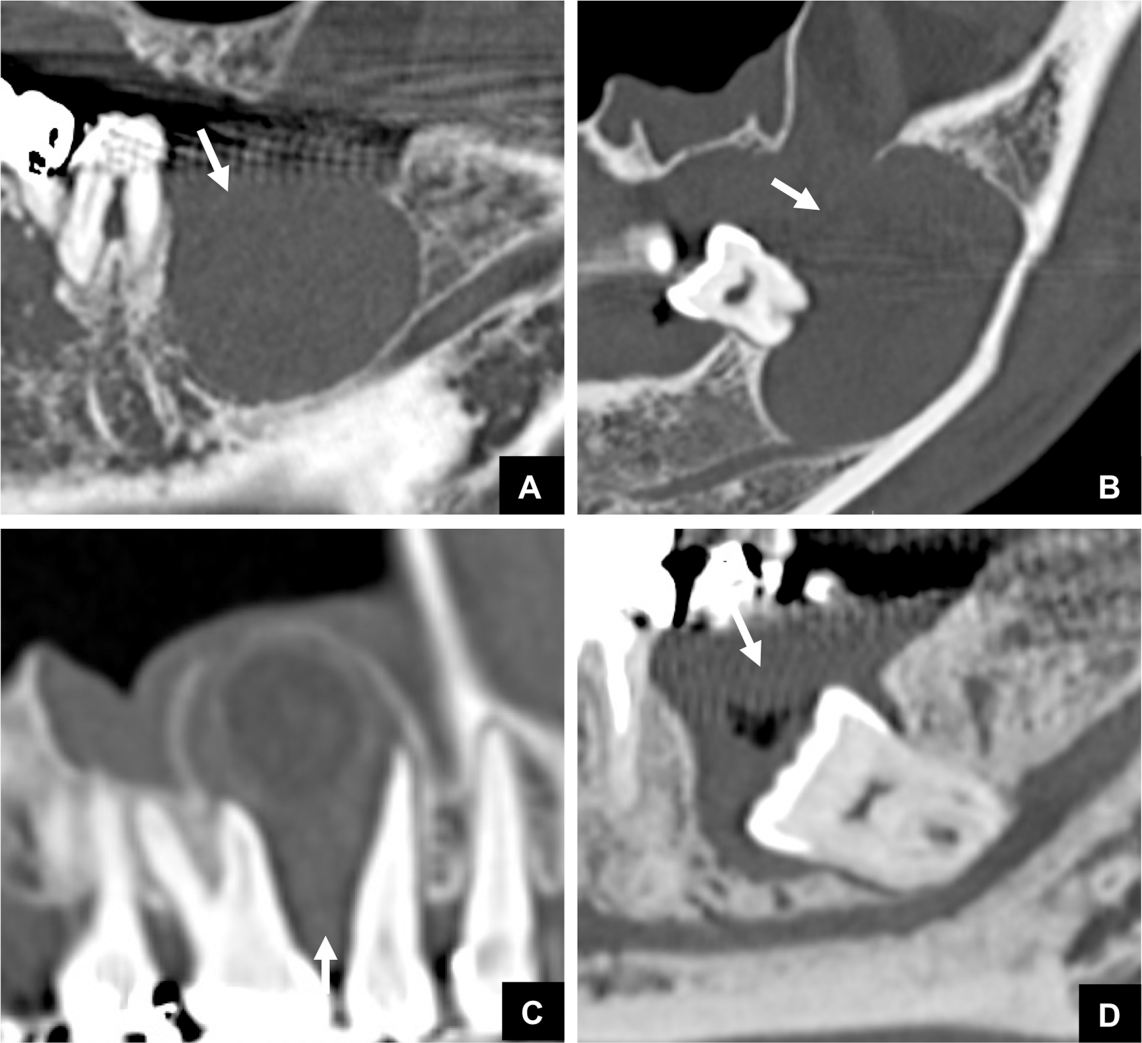
Representative MDCT images of the relationship between odontogenic masses and an expanded GT in cases classified as Group 2. Bone defect areas in the top of the alveolar bone that include a possibly expanded GT were detected (arrows). **(A)** MDCT image of an AM case classified as Group 2. **(B)** MDCT image of an OKC case classified as Group 2. **(C)** MDCT image of a COC case classified as Group 2. **(D)** MDCT image of a DC case classified as Group 2.

**Fig 4:**
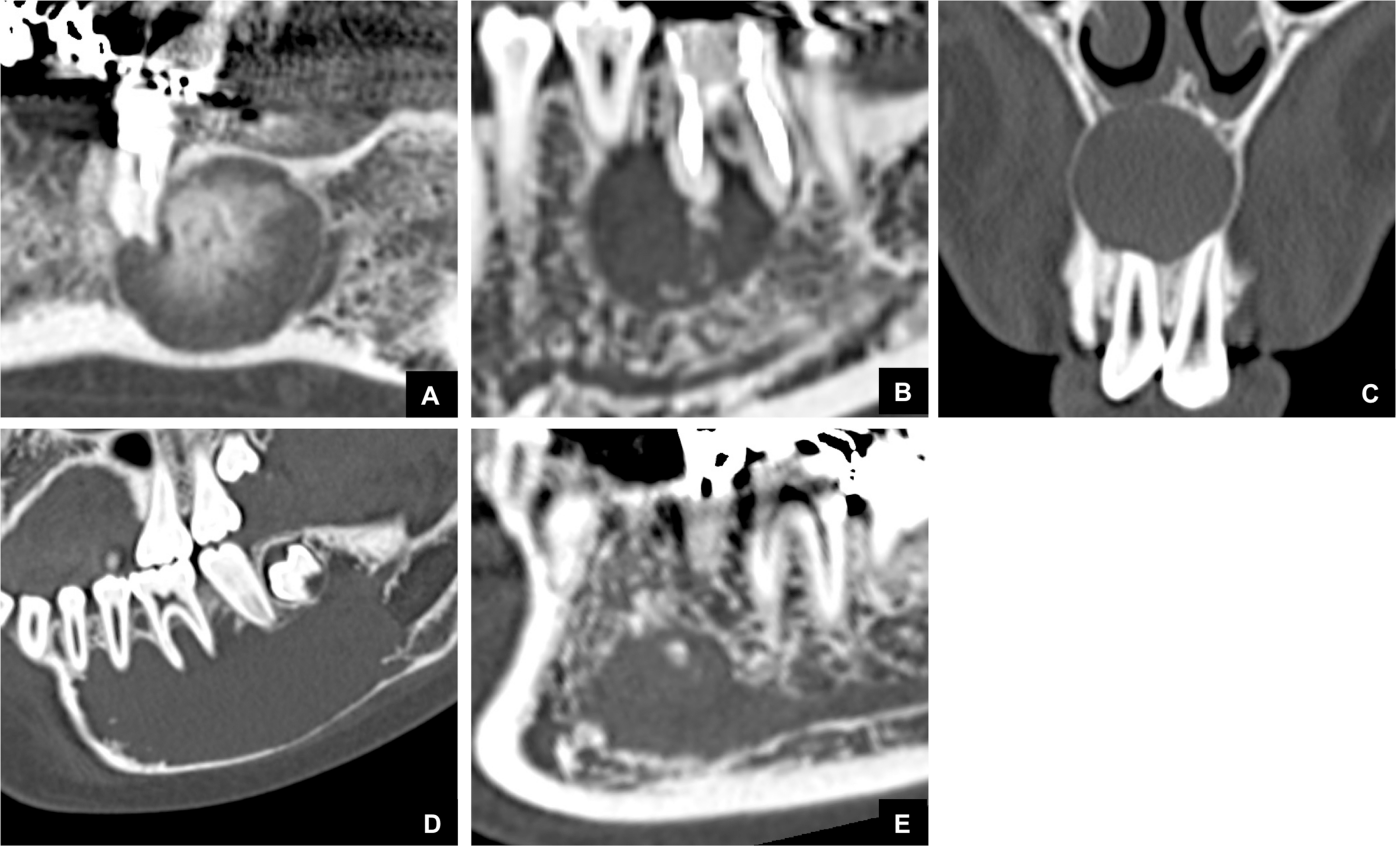
Representative MDCT images of the relationship between non-odontogenic masses and the GT in cases classified as Group 3. Bone defect areas at the top of the alveolar bone were not detected. **(A)** MDCT image of an ossifying fibroma case classified as Group 3. **(B)** MDCT image of a fibroma case classified as Group 3. **(C)** MDCT image of a nasopalatine duct cyst case classified as Group 3. **(D)** MDCT image of a simple bone cyst case classified as Group 3. **(E)** MDCT image of a schwannoma case classified as Group 3.

Cases were divided into three groups based on the continuity of the odontogenic or non-odontogenic mass with the GT as assessed by MDCT. The assignment of the sub-classifications of odontogenic or non-odontogenic masses to each group is shown in Table 2. Most cases of odontogenic tumors or cysts (239 cases) were included in Group 1 or Group 2, with Group 1 (191 cases) (Fig 2) including most of the cases of DC and OKC and Group 2 (48 cases) (Fig 3) including most of the cases of AM and COC. In contrast, almost all of the non-odontogenic tumors or cysts (57 cases) were included in Group 3 (Fig 4). This meant that, in most of the odontogenic tumors or cysts (239 cases), but not in the non-odontogenic tumors or cysts (one case), intact or expanded GTs were almost always detected on MDCT as low density tracts or low density areas that were contiguous both to the top of the alveolar bone and to the top of a tumor or cyst. The distribution of subjects with (Group 1 and 2) or without (Group 3) continuity area in odontogenic masses was compared to those with non-odontogenic masses by Fisher’s exact test. A significant difference was found on the imaging characteristics of the continuity associated with the GT between odontogenic and non-odontogenic masses (Fisher’s exact test; p = 0.0001).

**Table 2:** Continuity of each type of mass with the gubernaculum tract.

	Number of cases		
	Group 1	Group 2	Group 3
**Odontogenic masses**			
**Ameloblastomas**	5	14	2
**Odontogenic keratocysts**	34	12	4
**Calcifying odontogenic cysts**	-	2	-
**Dentigerous cysts**	152	20	10
**Total**	191	48	16
**Non-odontogenic masses**			
**Ossifying fibroma**	-	-	9
**Fibromas**	-	-	1
**Nasopalatine duct cysts**	-	1	25
**Simple bone cysts**	-	-	18
**Schwannomas**	-	-	4
**Total**	-	1	57

### Correlation between the presence or absence of, or the size of continuity areas and the sizes of odontogenic masses on MDCT

**Fig 5:**
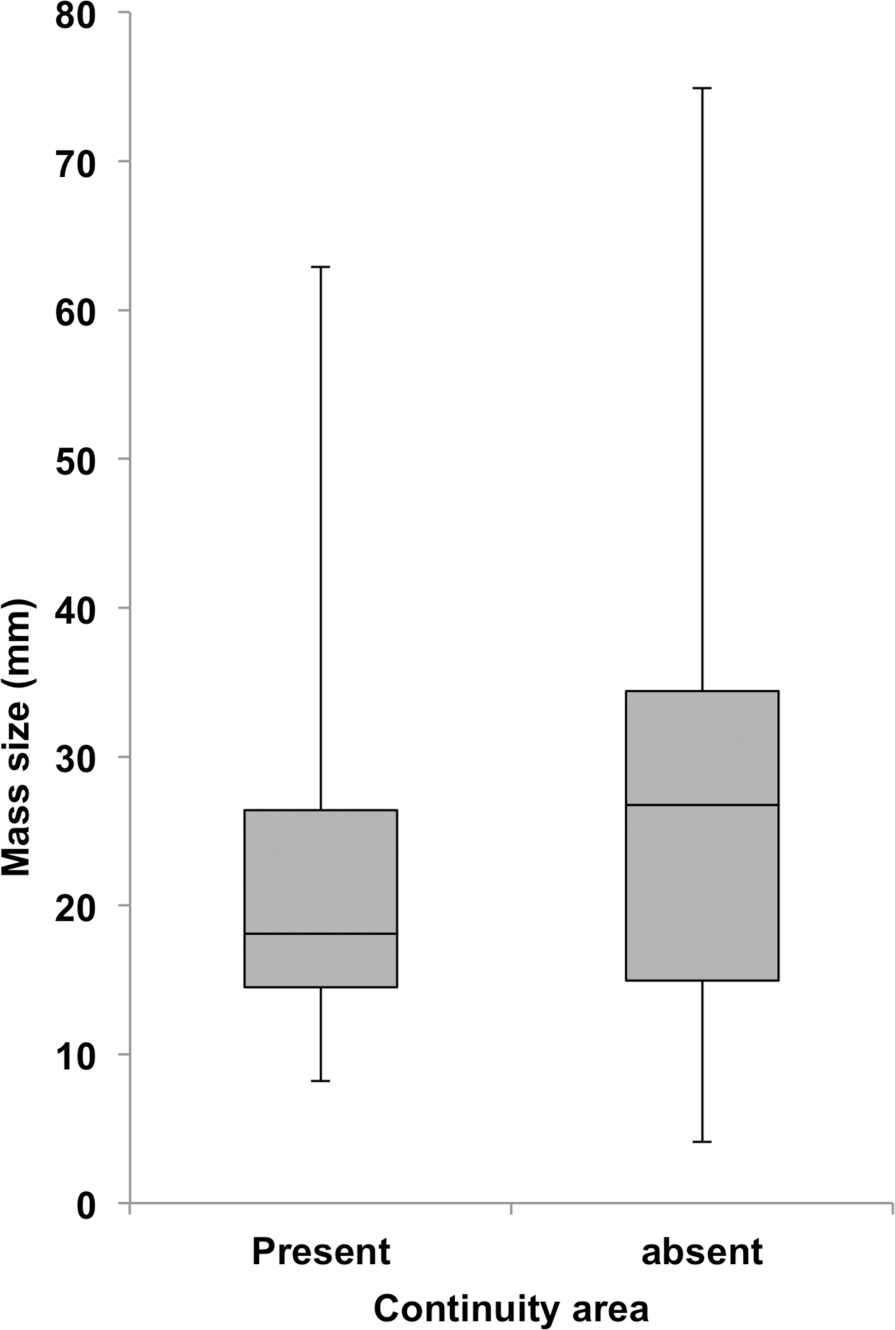
Box-and-whisker diagram of the sizes of the odontogenic masses in cases that do and do not have a continuity area in MDCT images.

**Fig 6:**
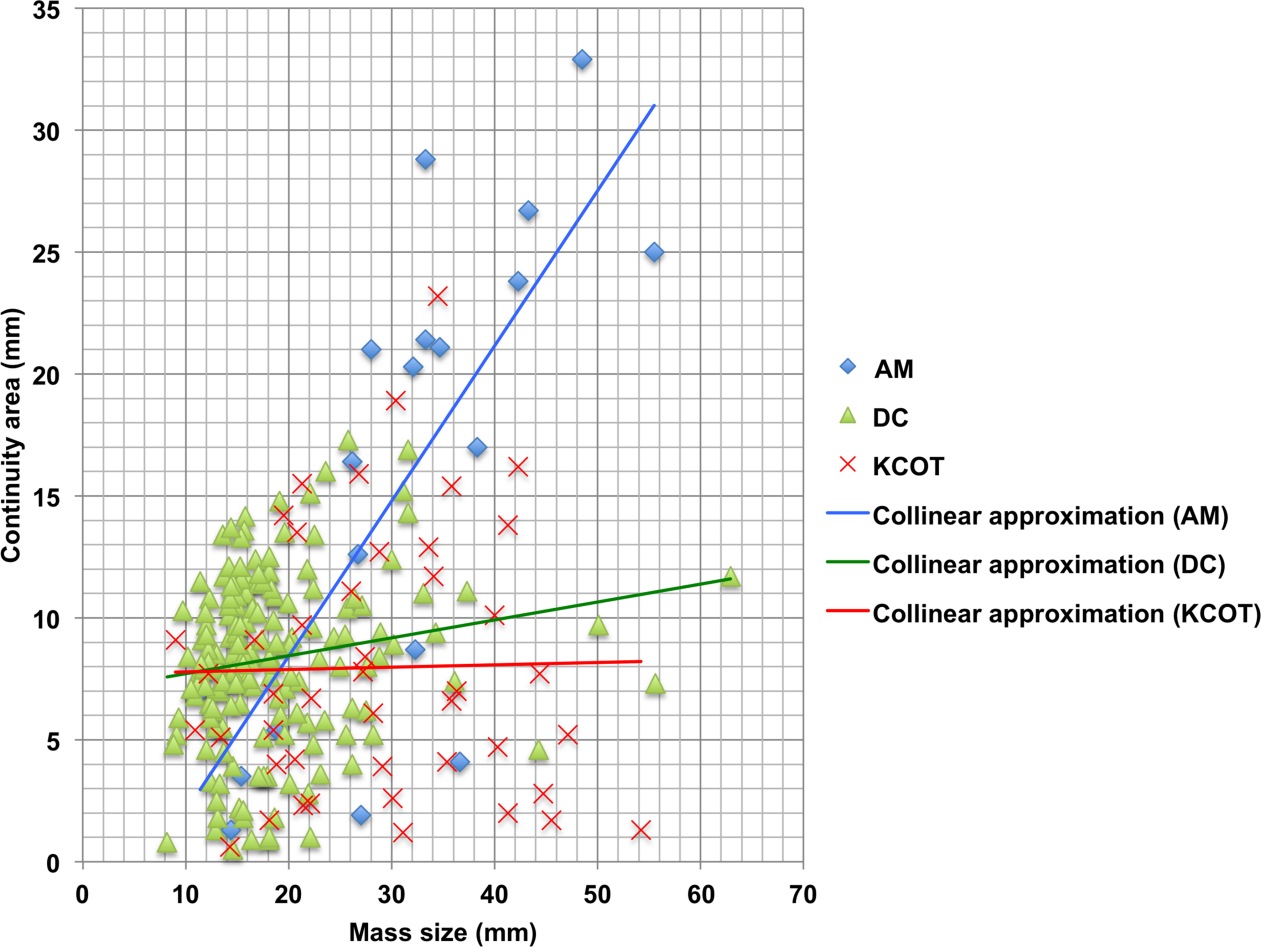
Relationship between mass size and area of the major axis of continuity on MDCT imaging in AM, DC and OKC cases classified as Group 1 or 2. In AM (blue diamonds), a strong significant correlation was found between the mass size and the size of the continuity area (r = 0.741, p = 0.0001). In DC (green triangles), a very weak significant correlation was found between the mass size and the size of the continuity area (r = 0.167, p = 0.0028). In OKC (red Xs), there was no correlation between the mass size and the size of the continuity area (r = -0.089, p = 0.557).

As the non-odontogenic and odontogenic masses were clearly divided into different groups with almost all non-odontogenic (98.2%) and only a few odontogenic masses (6.3%) belonging to Group 3, these MDCT findings regarding the continuity of masses with the GT could be used as the basis for differential diagnosis between odontogenic and non-odontogenic masses. Next, we analyzed the relationship between the alteration of continuity areas and the size of odontogenic masses on MDCT to determine if this information might be of further use for the differential diagnosis of odontogenic masses. No significant difference in the sizes of odontogenic masses was found between cases that had a continuity area (Group 1 and 2; 239 cases) and those that did not (Group 3; 16 cases) by t-test (p = 0.170) (Fig 5) (Table 3). However, when the odontogenic masses that had the continuity area (Group 1 and 2) were subdivided into the three representative types of odontogenic masses (AM, DC, OKC), each type showed a characteristic correlation between the mass size and the alteration of the continuity area (Fig 6). Thus, the correlation between the mass size and the size of the continuity area was strong for AM (r = 0.741, p = 0.0001), with the continuity area increasing along with the size of the AM mass by Pearson’s correlation coefficient. This correlation was very weak for DC (r = 0.167, p = 0.0028) and there was no significant correlation between mass size and continuity area size in OKC (r = -0.089, p = 0.557).

**Table 3:** Odontogenic mass size distribution in the presence or absence of the continuity area.

	Number of cases		
Mass size (mm)	Present	Absent	Total
**-10**	6	1	7
**10–20**	136	6	142
**20–30**	51	3	54
**30–40**	26	3	29
**40–50**	15	1	16
**50–60**	4	1	5
**60–70**	1	-	1
**70–80**	-	1	1
**Total**	239	16	255

### Analysis of cases of odontogenic tumors or cysts that were classified as having no GT and no continuity area (Group 3) on MDCT

**Fig 7:**
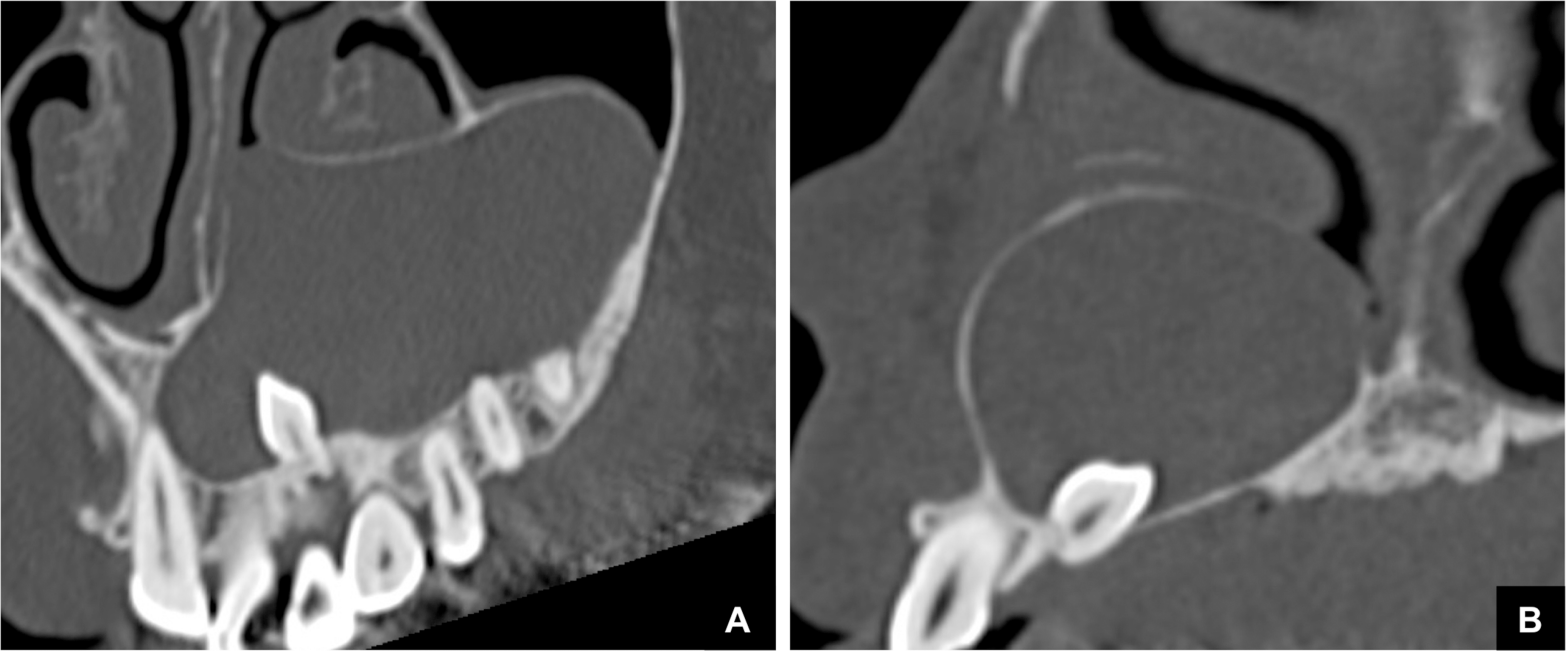
Representative MDCT images of DC classified as Group 3. **(A)** Panoramic MDCT image. A GT cannot be detected at the top of the DC. The DC was related to, and derived from the supernumerary teeth. **(B)** Cross sectional MDCT image of Fig 7A.

**Fig 8:**
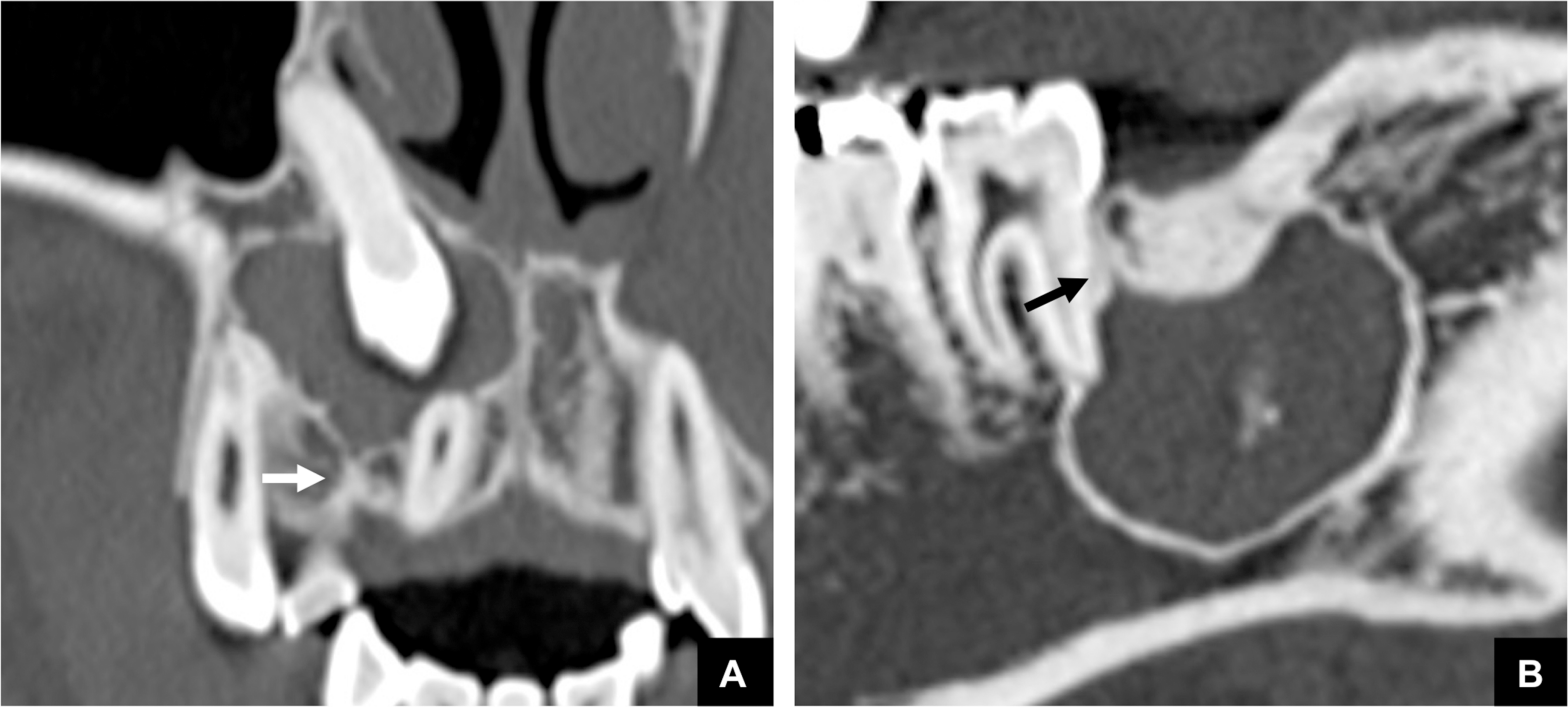
Representative MDCT images of DC and OKC classified as Group 3, with a calcified duct-like structure. A calcified duct-like structure, which may represent possible remnants, was visualized at the top of a DC (arrow) **(A)** and at the top of an OKC (arrow) **(B)**. The structure continued from the DC or OKC, and ran along with a typical area of the GT.

There were16 cases with odontogenic tumors or cysts that were classified as Group 3, i.e., as having no GT and no continuity area on MDCT (Table 2). These cases comprised 10 cases of DC, 4 of OKC, and 2 of AM. Five of these 10 DC cases were related to, and derived from the supernumerary teeth (Fig 7). Calcified duct-like structures, which may possibly be remnants, were visualized at the top of the masses in 3 of the 10 cases of DC and in 3 of the 4 cases of OKC (Fig 8). However, apart from these findings, no other imaging findings were noted.

## Discussion

Previously, a clear anatomic association has been shown between the GT and AOTs on CT [5], and we suggested that the GT might be associated with the origin of odontomas. Additionally, it has been suggested that the pathogenesis of AM and OKC may be related to the GT [7, 8]. We therefore hypothesized that the imaging characteristics of GTs might be useful for the diagnosis of odontogenic tumors or cysts. To investigate this possibility, in the present study, we determined the imaging characteristics of various odontogenic and non-odontogenic masses on CT and analyzed their relationship with the GT.

The most interesting result of the present study is that continuity of a mass with the GT could be detected on CT for almost all of the odontogenic masses (93.7%), but could not be detected for the non-odontogenic masses. This result means that the odontogenic/non-odontogenic character of a mass can be diagnosed based on analysis of the continuity of the mass with the GT. This is the first time this type of diagnosis has been reported and this result is of great significance for the diagnosis of diseases in the oral and maxillofacial region. One possible reason as to why this very important finding has not been previously discovered may be because little attention has been paid to the GT by people working in medical and dental fields. Thus, when diagnosing odontogenic masses, the significance of the image findings of the GT in relationship to odontogenic masses, including the possibility that the origin of the odontogenic mass might be the GT, was not taken into consideration.

Another important result of this study was that there was no significant difference between the sizes of masses that did and did not have a continuity area. The significance of this result is that even small odontogenic masses continue to the top of alveolar bone. If only the larger odontogenic masses had the characteristic image finding mentioned above, the present result could be meaningless. Based on the combined data together with previous reports [2, 5, 7, 8, 9], we speculate that the origin of almost all odontogenic masses may be partly associated with the GT. Our first opinion based on the present precise analysis on MDCT introduces a new era in the diagnosis of these masses and may potentially alter the classification of odontogenic masses.

A second interesting result of this study was the characteristic correlation between the mass size and the size of the continuity area in the 3 representative types of odontogenic masses. Thus, whereas a highly significant correlation was found between mass size and the size of the continuity area in AM, where the continuity area increased along with the size of the AM, this correlation was very weakly significant in DC. These data meant that it was possible to differentially diagnose the mass as AM or DC based on image findings regarding the size of the continuity area. However, as there was no correlation between the mass size and the size of the continuity area in OKC, this made it difficult to differentially diagnose between AM, DC and OKC. At present, we cannot explain this result, but we expect that further study will clarify this matter.

Another interesting finding was obtained in the analysis of cases with odontogenic tumors or cysts that were classified as no GT and no continuity areas, in which we identified calcified duct-like structures at the top of masses that may be possible remnants. The present imaging finding was the first and quite reasonable detection on GT, and sustained our present suggestion. However, these structures could not be identified on pathological specimens. Further study may help to clarify this matter. In addition, in most of the DC cases that were classified as Group 3, the DC were related to and derived from the supernumerary teeth. We previously reported that it may be difficult to identify the GT in supernumerary teeth [1, 6]. However, this explanation for the lack of detection of a GT would not be applicable to all cases without a GT and further study is required regarding this point.

Regarding the diagnosis of odontogenic masses, based on both the present and previous findings, we suggest that the presence of and contact with the GT should be added as a characteristic CT finding of some types of odontogenic masses including DC, COC, OKC, AM, AOT, odontomas, and perhaps others [2, 5]. In particular, as an intact GT was present in most DC, COC, and odontomas, the presence or absence of the GT could be a useful finding when differentially diagnosing DC and OKC from AM.

The limitations of this study are as follows. First, as this was a retrospective study, the pathological specimens were prepared for the diagnosis of odontogenic and non-odontogenic masses, and not for evaluation of the GT. This meant that we were unable to analyze the pathological findings of GT. Preparation of pathological specimens for evaluation of the relationship between the GT and odontogenic or non-odontogenic masses is clearly difficult because the maxilla and mandible consist of hard tissue, but the GC does not. In addition, the present results should be interpreted as being relevant to relatively healthy, active populations. A further limitation of this study was that only Japanese subjects were examined.

In conclusion, we believe that, based on our data, dentists should pay more attention to the association between the GT and odontogenic masses. We expect that many more studies regarding this association will be performed in the future.
